# The long non-coding RNA LINC00473 contributes to cell proliferation via JAK-STAT3 signaling pathway by regulating miR-195-5p/SEPT2 axis in prostate cancer

**DOI:** 10.1042/BSR20191850

**Published:** 2020-09-16

**Authors:** Zengshu Xing, Sailian Li, Zhenxiang Liu, Chong Zhang, Meijiang Meng, Zhiming Bai

**Affiliations:** 1 Department of Urology, Affiliated Haikou Hospital of Xiangya Medical College, Central South University, No.43, Renmin Road, Meilan District, Haikou, 570208, Hainan Province, P.R. China; 2 Department of Gastroenterology, Affiliated Haikou Hospital of Xiangya Medical College, Central South University, Haikou 570208, Hainan Province, P.R. China

**Keywords:** JAK-STAT3 signaling pathway, LINC00473, miR-195-5p, prostate cancer, SEPT2

## Abstract

Prostate cancer is a kind of male malignant tumor, which has brought tremendous health threat to men. Prostate cancer is difficult to be cured because of high incidence and metastasis rate. Thereby, it is of great urgency to elucidate the underlying molecular mechanism of prostate cancer for the treatment of this cancer. LINC00473 dysregulation has been observed in many cancers. However, the role of LINC00473 was unknown in prostate cancer. In the present study, we discovered that prostate cancer cells presented high expression of LINC00473, and LINC00473 inhibition limited cell proliferation and the expression of proteins in JAK-STAT3 signaling pathway. Additionally, LINC00473 acted as an up-stream factor for miR-195-5p to negatively modulate miR-195-5p expression. Moreover, SEPT2 interacted with miR-195-5p in prostate cancer and SEPT2 expression was positively modulated by LINC00473 and negatively regulated by miR-195-5p. Last, the inhibitory effect of LINC00473 knockdown on cell proliferation and expression of proteins of JAK-STAT3 signaling pathway was restored by SEPT2 overexpression. All in all, LINC00473 contributed to cell proliferation via JAK-STAT3 signaling pathway by regulating miR-195-5p/SEPT2 axis in prostate cancer, which provided a novel therapeutic tactic for prostate cancer patients.

## Introduction

Prostate cancer is a kind of male malignant tumor [1], accounting for approximately 20% of new cases of cancers in males, which has brought tremendous health threat to men globally [2]. With the highest incidence for men, prostate cancer ranks as the second most lethal cancers among men, second only to lung cancer [3]. Known risk factors including diet, advanced age, sexual intercourse and genetic factors have been discovered [4]. Androgen deprivation therapy and radiotherapy are main treatments for the early stage prostate cancer [2]. However, in spite of the initial success in taking control of tumor progression [5], the underlying mechanism and pathogenesis of prostate cancer remain elusive. Therefore, it is of great urgency to figure out the underlying mechanism of prostate cancer.

Long noncoding RNAs (lncRNAs) are a category of endogenous cellular RNAs with more than 200 nucleotides, without the capacity to encode proteins [6,7]. Increasing reports demonstrated that the aberrant expression of lncRNAs exert tumor-promoting or tumor-suppressive effects on different types of cancers [8,9]. Moreover, recent evidence revealed that lncRNAs can be involved in the regulation of several cellular processes, such as cell proliferation, cell cycle, differentiation and apoptosis [10,11]. For example, long noncoding RNA LINC00261 suppresses cell proliferation and invasion and promotes cell apoptosis in human choriocarcinoma [12]. Down-regulation of long non-coding RNA AFAP1-AS1 inhibits tumor growth, promotes apoptosis and decreases metastasis in thyroid cancer [13]. LINC00473 has been proved to play an oncogenic role by sponging microRNA in diverse cancers including breast cancer, head and neck squamous cell carcinoma and colorectal cancer [14–16]. For instance, LINC00473 displays a poor prognosis and drives tumorigenesis of breast cancer by sponging miR-497 [17]. LINC00473 facilitates the Taxol resistance by sponging miR-15a in colorectal cancer [16]. However, the roles of LINC00473 were still unclear in prostate cancer.

Abundant evidences verified that lncRNAs could act as a competing endogenous RNA (ceRNA) by sponging microRNAs (miRNAs) to regulate the expression of target genes [18]. SEPT2 has been proved to drive hepatoma cell growth [19], and subsequently further explored by increasing research. For instance, SEPT2 facilitates cell migration and invasion by activating MEK/ERK in breast cancer [20]. SEPT2 functions as a target gene of mir-140-5p to drive the development of biliary tract cancer [21]. However, SEPT2 serving as a target gene in prostate cancer has not been explored yet.

In our research, we explored the role of LINC00473 and the regulation mechanism between LINC00473 and the JAK-STAT3 signaling pathway in prostate cancer. Our results suggested that LINC00473 contributes to cell proliferation via JAK-STAT3 signaling pathway by regulating miR-195-5p/SEPT2 axis in prostate cancer.

## Materials and methods

### Cell culture and cell transfection

For cell culture, prostate cancer cells (DU145, LNCaP and PC-3) human normal prostate epithelial cells (P69) were purchased from CELLCOOK organization (Guangzhou, China). Cells were cultured in DMEM medium (Gibco) supplemented with 10% fetal bovine serum (FBS) (Gibco) and 1% penicillin or streptomycin (Gibco). Cells were cultured at 37°C in a humid air with 5% CO_2_. For cell transfection, shRNAs targeting LINC00473 (sh-LINC00473#1, sh-LINC00473#2 and sh-LINC00473#3) were used to knockdown LINC00473 expression with sh-NC as scramble control. MiR-195-5p mimics were adopted to amplify miR-195-5p expression with NC mimics as internal control. SEPT2 expression was up-regulated by pcDNA3.1/SEPT2 transfection normalized by the transfection of pcDNA3.1. Vectors used in this assay were purchased from GenePharma (Shanghai, China). According to the manufacturer’s instruction, all transfections were carried out using Lipofectamine 2000 (Invitrogen, Carlsbad, CA, U.S.A.).

### Real-time reverse-transcription polymerase chain reaction (RT-qPCR)

Total RNA extraction using prostate cells was operated in TRIzol reagent (Invitrogen, U.S.A.), and then using M-MLV Reverse Transcriptase Kit (Invitrogen, U.S.A.) was employed to reversely transcribe RNA into the first strand of cDNA. Real-time PCR analyses were carried out in triplicate for each sample using SYBR Green PCR Master Mix (TOYOBO) on a LightCycler 480 system (Roche). Glyceraldehyde-3-phosphate dehydrogenase (GAPDH) served as the endogenous control. For detecting miRNA expression level, cDNA was synthesized using a TaqMan® miRNA reverse transcription kit (Applied Biosystems, Foster City, CA, U.S.A.), and U6 small nuclear RNA served as the endogenous control. All primers are listed below:

LINC00473: forward, 5′-GATGGAAAGGAGGGAAGG-3′ and reverse, 5′-CACAGTGGGTCCAGGGTT-3′;
MiR-195-5p: forward, 5′-ACACTCCAGCTGGGTAGCAGCACAGAAAT-3′ and reverse, 5′-TGGTGTCGTGGAGTCG-3′;
SEPT2: forward, 5′-GGTGACGCTATCAACTGCAGAG-3′ and reverse, 5′-ATGATGTGCCGCCTGTTCAAGC-3′;
GAPDH: forward, 5′-GAAGGTGAAGGTCGGAGTC-3′ and reverse, 5′-GAAGATGGTGATGGGATTTC-3′;
U6: forward, 5′-ATTGGAACGATACAGAGAAGATT-3′ and reverse, 5′-GGAACGCTTCACGAATTTG-3′.

### 5-ethynyl-2′-deoxyuridine (EdU) assay

PC3 and DU145 cells were incubated in EdU solution for 2 h. Then, the cells were deposited in 70% ethanol, after being fixed with PBS containing 4% paraformaldehyde. Finally, the cells were stained by Cell-Light^™^ EdU Apollo®488 In Vitro Imaging Kit (RioBio, China). Cell growth was observed by Fluorescence microscopy.

### Colony formation assay

After 48 h of transfection, cells were trypsinized into single-cell status. Then, these cells were grown in a 6-well plate at a density of 800 cells per well and cultivated for 2 weeks at 37°C. After the incubation, the plate was gently rinsed with 10% PBS. The cells were immobilized with 1% methanol solution, and then dyed with 0.1% Crystal Violet. Colonies over 50 cells were observed and manually counted.

### Western blot analysis

Cells were lysed with the help of RIPA lysis buffer (Beyotime Biotechnology, China). Total protein extracts were separated by 10% SDS-PAGE and immediately transferred onto PVDF membranes (GE Healthcare Bio-Sciences Corp., Piscataway, NJ, U.S.A.). Afterward, the membranes were incubated using primary antibodies of JAK2 (ab108596, 1:5000), p-JAK2 (ab32101, 1:1000), STAT3 (ab68153, 1:1000), p‐STAT3 (ab76315, 1:2000), PI3K (ab32089, 1:1000), p-PI3K (ab182651, 1/500), T-AKT (ab32505, 1/2000), p-AKT (ab38449, 1/500), I-κB (ab32518, 1/10000), p-I-κB (ab133462, 1/10000), SHH (ab53281, 1/1000), PTCH1 (ab53715, 1/500), GLI1 (ab49314, 1/500), GAPDH (ab9485, 1:2500). All primary antibodies were purchased from Abcam Company (Abcam, Cambridge, U.K.). Then, the membranes were appropriately incubated using secondary antibodies. Finally, blots were imaged by ECL detection reagents (Amersham Biosciences, Sweden).

### RNA immunoprecipitation (RIP) assay

To confirm whether miR-195-5p could bind with LINC00473, magna RNA immunoprecipitation (RIP) kit (Millipore, Billerica, U.S.A.) was adopted. Magnetic beads containing Ago2 or IgG (negative control) antibodies were added into cell lysate that was preserved in RIP buffer before. The relative expression of miR-195-5p and LINC00473 were detected by RT-qPCR assay.

### Luciferase reporter assay

The wild-type (WT) and mutant (Mut) full-length of LINC00473 (LINC00473-WT/LINC00473-Mut) or WT and Mut 3′-UTR (untranslated region) sequences of SEPT2 (SEPT2-WT/SEPT2-Mut) were constructed into pmirGLO vectors. Then, the constructed plasmids were co-transfected with miR-195-5p mimics or NC mimics into PC3 and DU245 cells by Lipofectamine® 2000 reagent (Invitrogen). According to the manufacturer’s protocols, luciferase activity was evaluated by the Dual-luciferase Reporter Assay System (Promega, Madison, WI, U.S.A.).

### RNA pull-down assay

Biotinylated miR-195-5p probes were co-cultivated with cell lysate (PC3 andDU145) at 4°C overnight, with the no-biotin labeled probes as negative controls. The probes were synthesized by Genechem (Shanghai China). After purifying, elution buffer was used to elute RNAs and then RT-qPCR was performed to examine the enrichment of LINC00473 and SEPT2 in compounds obtained from different groups.

### Nuclear–cytoplasmic fractionation

The location of LINC00473 in cytoplasm or nucleus of PC3 and DU145 cells was determined with the application of a PARIS kit (Life Technologies, MA, U.S.A.). Briefly, cells were collected and lysed on ice. After centrifugation, the supernatant was harvested. The extracted RNAs were tested by RT-qPCR, while GAPDH and U6 were utilized as the cytoplasmic and nuclear controls, respectively.

### Statistical analysis

Statistics were analyzed utilizing the SPSS 16.0 software system (SPSS, Chicago, IL). The experiments mentioned were all conducted in triplicate and all data were expressed taking the form of the mean ± standard deviation (SD). Student’s *t*-test or one-way analysis of variance (ANOVA) was employed to evaluate differences between two groups or more than two groups. *P* < 0.05 was regarded to be statistically significant.

## Result

### LINC00473 presents high expression to aggravate prostate cancer via the JAK/STAT3 signaling pathway

**Figure 1:**
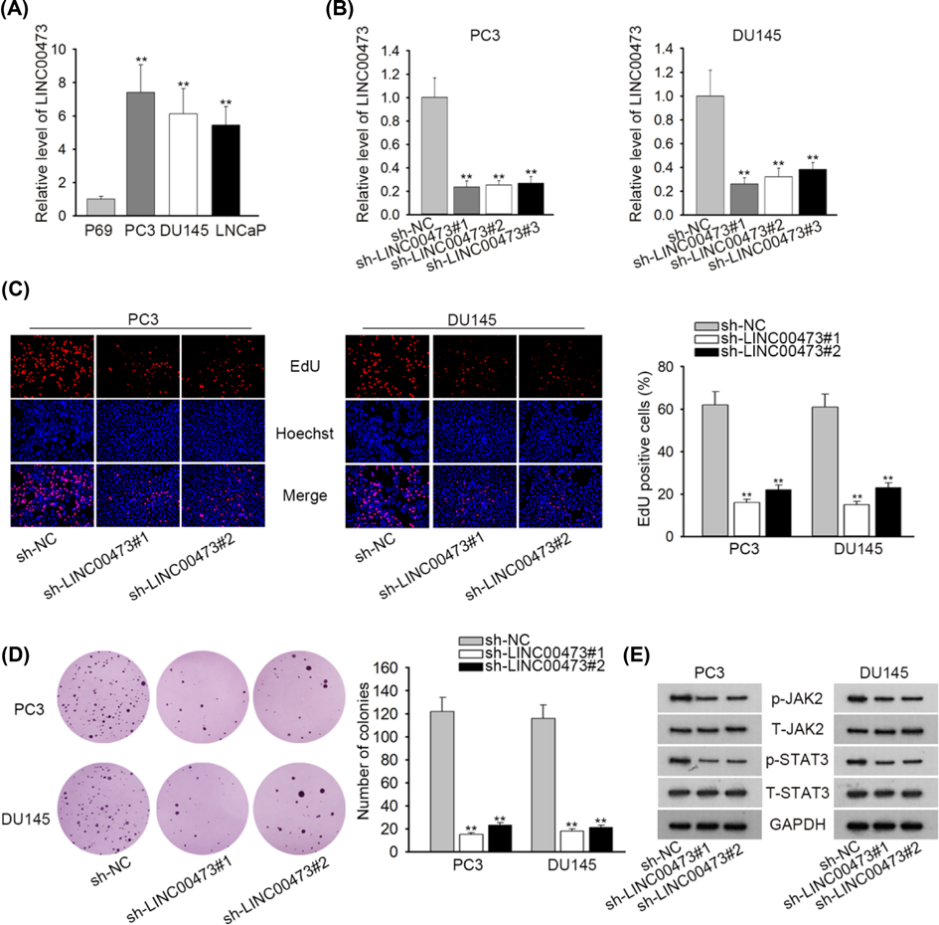
LINC00473 presented high expression to aggravate prostate cancer via JAK/STAT3 signaling pathway (**A**) LINC00473 expression was measured by RT-qPCR assay in prostate cancer cells (PC3, DU145 and LNCaP) and human normal prostate epithelial cells (P69). (**B**) The knockdown efficiency of LINC00473 was assessed by RT-qPCR assay. (**C** and **D**) The proliferative capability of prostate cancer cells was evaluated by EdU and colony formation assays. (**E**) Western blot analysis was performed to detect the protein expression of JAK2, STAT3, phosphorylated JAK2 and STAT3; **P<0.01.

The high expression of LINC00473 has been observed in a couple of cancers including breast cancer, esophageal squamous cell carcinoma and colorectal cancer [16,17,22], while the expression of LINC00473 in prostate cancer remains unknown. The result of RT-qPCR assay showed that LINC00473 expression was higher in prostate cancer cells (PC3, DU145 and LNCaP) than that in human normal prostate epithelial cells (P69) (Figure 1A). To explore the biological function of LINC00473, sh-LINC00473#1/2/3 was separately transfected into PC3 and DU145 cells, which resulted in an evident decrease in LINC00473 expression compared with scramble control (Figure 1B). Moreover, sh-LINC00473#1 and sh-LINC00473#2 displayed better inhibition efficiency than sh-LINC00473#3 and were chosen for following assays. At first, EdU assay indicated that the knockdown of LINC00473 triggered an evident decrease in EdU positive PC3 and DU145 cells (Figure 1C). Similarly, the number of colonies was reduced by transfecting sh-LINC00473#1 or sh-LINC00473#2 into PC3 and DU145 cells as well (Figure 1D). Then, we sought to examine the downstream signaling pathways of LINC00473. Western blot analysis was applied to examine several pathways including PI3K/Akt pathway, Hedgehog pathway, JAK-STAT3 pathway and NF-κB pathway in cells transfected with sh-LINC00473#1/2. The results depicted that LINC00473 had no regulatory effects on PI3K/Akt pathway, Hedgehog pathway and NF-κB pathway (Supplementary Figure S1A). Importantly, we first discovered that transfection of sh-LINC00473#1 or sh-LINC00473#2 caused reduction of the protein expression of phosphorylated JAK2 and STAT3, while that of total JAK2 and STAT3 was nearly unchanged (Figure 1E), indicating that LINC00473 activated JAK-STAT3 pathway. Collectively, up-regulated LINC00473 in prostate cancer facilitates cell proliferation and activates JAK/STAT3 signaling pathway.

### LINC00473 interacts with miR-195-5p in prostate cancer

**Figure 2:**
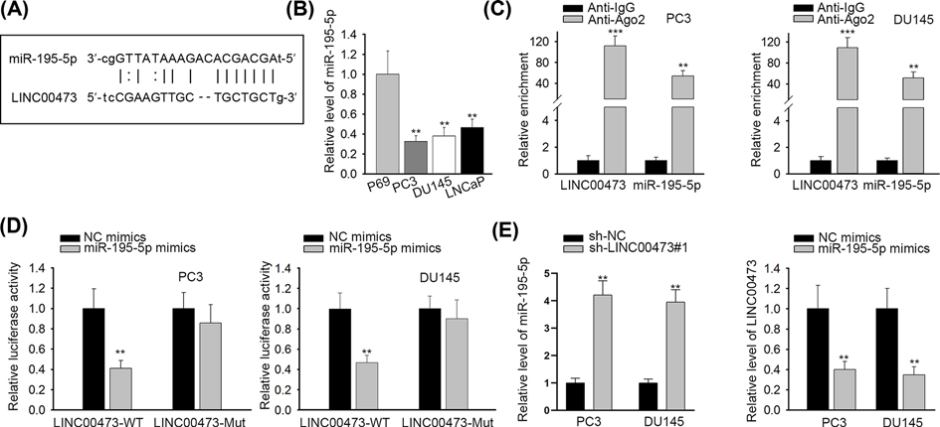
LINC00473 interacted with miR-195-5p in prostate cancer (**A**) The binding sites between LINC00473 and miR-195-5p were predicted by starBase. (**B**) RT-qPCR assay was operated to examine the expression of miR-195-5p. (**C** and **D**) The binding capacity between LINC00473 and miR-195-5p was validated by RIP and luciferase reporter assay. (**E**) RT-qPCR assay was carried out to test the interaction between LINC00473 and miR-195-5p; **P<0.01, ***P<0.001.

To deeply explore the molecular mechanism of LINC00473 administrating proliferation of prostate cancer cells, starBase (http://starbase.sysu.edu.cn) was employed to seek putative miRNA that could bind to LINC00473. As shown in Figure 2A, miR-195-5p had several binding sites for LINC00473. Additionally, compared with normal prostate cells (P69), prostate cancer cells indicated lower expression of miR-195-5p (Figure 2B). According to RIP assay, not only LINC00473 but also miR-195-5p was abundant in anti-Ago2 group rather than anti-IgG group (Figure 2C), delineating that LINC00473 and miR-195-5p coexisted in RNA-induced silencing complexes (RISCs). Luciferase reporter assay using PC3 and DU145 cells demonstrated that miR-195-5p amplification led to an obvious attenuation of luciferase activity in LINC00473-WT vectors and no alteration was noticed in LINC00473-Mut vectors (Figure 2D). Finally, miR-195-5p expression was decreased by LINC00473 inhibition and miR-195-5p overexpression down-regulated LINC00473 expression (Figure 2E). To conclude, LINC00473 interacts with miR-195-5p and negatively modulates miR-195-5p expression.

### SEPT2 is a target gene of miR-195-5p in prostate cancer

**Figure 3:**
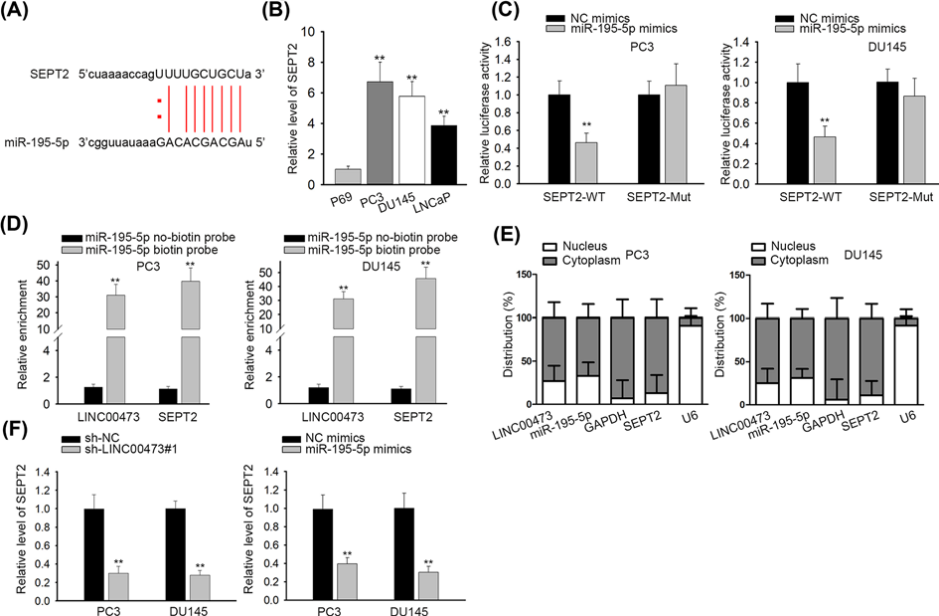
SEPT2 was a target gene of miR-195-5p in prostate cancer (**A**) The binding sites between miR-195-5p and SEPT2 were predicted by starBase. (**B**) RT-qPCR assay was operated to detect the expression of SEPT2. (**C**) Luciferase reporter assay was adopted to confirm the binding capability between miR-195-5p and SEPT2. (**D**) RNA pull down assay verified the binding abilities between miR-195-5p and LINC00473 (or SEPT2) (**E**) Nuclear–cytoplasmic fractionation assay was used to detect the subcellular location of LINC00473, miR-195-5p and SEPT2. (**F**) RT-qPCR assay was employed to test SEPT2 expression; **P<0.01.

Abundant reports proposed that miRNAs could modulate expression of target genes by specifically binding with the 3′UTR of target genes [23,24], so we predicted that miR-195-5p also worked in this pattern in prostate cancer. Based on the prediction of starBase, SEPT2 was discovered to combine with miR-195-5p (Figure 3A). Then, prostate cancer cells (PC3, DU145 and LNCaP) displayed higher SEPT2 expression than P69 cells (Figure 3B). The result of luciferase reporter assay elucidated that the luciferase activity of SEPT-WT vectors in PC3 and DU145 cells was attenuated by transfection of miR-195-5p mimics whereas no apparent changes of luciferase activity were observed in SEPT2-Mut group (Figure 3C). RNA pull down assay depicted that both LINC00473 and SEPT2 were enriched by biotin-labeled miR-195-5p probe but not by no-biotin miR-195-5p probe (Figure 3D). Nuclear–cytoplasmic fractionation assay indicated that LINC00473, miR-195-5p and SEPT2 were all predominantly located in cytoplasm (Figure 3E). Either LINC00473 inhibition or miR-195-5p overexpression decreased SEPT2 expression (Figure 3F). To sum up, SEPT2 is the target gene downstream of LINC00473/miR-195-5p pathway in prostate cancer.

### LINC00473 contributes to cell proliferation via JAK-STAT3 signaling pathway by regulating miR-195-5p /SEPT2 axis in prostate cancer

**Figure 4:**
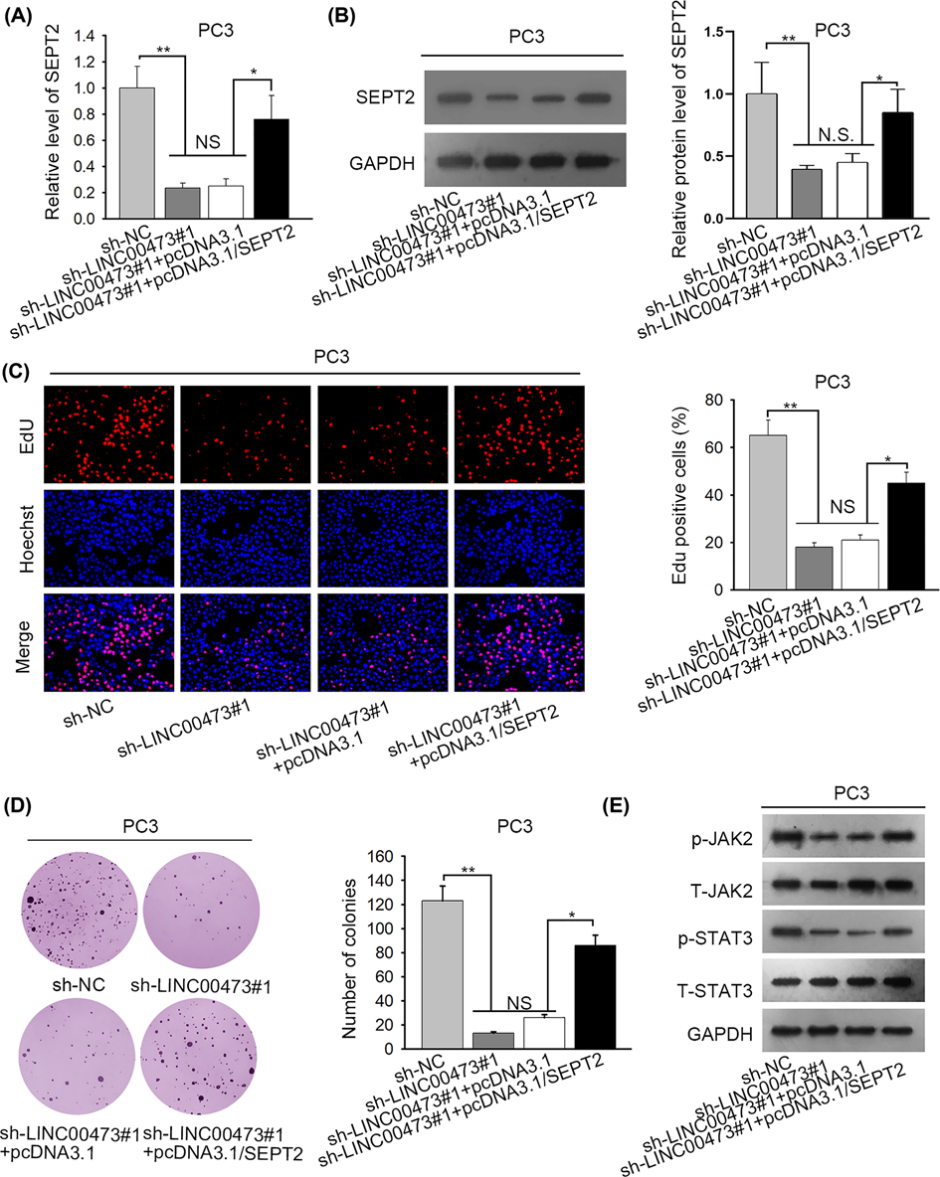
LINC00473 contributed to cell proliferation via JAK-STAT3 signaling pathway by regulating miR-195-5p /SEPT2 axis in prostate cancer (**A**) RT-qPCR assay showed that SEPT2 rescued the expression of SEPT2. (**B**) Western blot analysis was operated to monitor the protein expression of SEPT2. (**C** and **D**) EdU and colony formation assays were implemented to investigate cell proliferation. (**E**) The protein expression of JAK2, STAT3, phosphorylated JAK2 and STAT3 was determined by Western blot analysis; *P<0.05, **P<0.01, NS indicated no significance.

To validate whether LINC00473 exerted the oncogenic impacts on prostate cancer via miR-195-5p/SEPT2 axis, rescue assays were performed. At the beginning, the down-regulation of mRNA and protein expression of SEPT2 resulting from LINC00473 suppression was reversed by overexpressing SEPT2 (Figure 4A,B). Subsequently, EdU and colony formation assays using PC3 cells uncovered that the inhibitive effect on proliferative capability of LINC00473 silence was partially rescued by transfection of pcDNA3.1/SEPT2 (Figure 4C,D). Last but not least, the protein expression of phosphorylated JAK2 and STAT3 down-regulated by LINC00473 knockdown was restored by SEPT2 overexpression (Figure 4E). In a word, LINC00473 contributes to cell proliferation via JAK-STAT3 signaling pathway by regulating miR-195-5p/SEPT2 axis in prostate cancer.

## Discussion

Prostate cancer is deemed as a common malignant tumor in men, which has contributed to millions of deaths in the past. Investigating the therapeutic tactics is of great significance to find treatment for prostate cancer. Recent studies propose that several lncRNAs including LOXL1-AS1, PVT1, and HOTAIR exhibit abnormal expression in prostate cancer. These lncRNAs intricately participate in tumor progression, such as cell proliferation, cell migration and cell apoptosis. Our studies demonstrated that LINC00473 was involved in the modulation of cell proliferation and the expression of proteins in JAK-STAT3 signaling pathway.

LncRNAs dysregulation was closely associated with the pathogenesis and development of various diseases and cancers. Moreover, lncRNAs play a significant role in the regulation of a series of cellular process. Similarly, in our exploration, LINC00473 was observed to be highly expressed in prostate cancer cells. Moreover, LINC00473 inhibition significantly hampered the proliferative capability of prostate cells and inactivated JAK-STAT3 signaling pathway. LncRNAs were also reported to specifically bind with miRNAs to modulate tumor progression [25]. In present study, LINC00473 specifically bound with miR-195-5p. Additionally, miR-195-5p expression was negatively modulated by LINC00473. A previous study also reported that LINC00473 sponged miR-195-5p, and drove the progression of pancreatic cancer [26]. The previous study revealed that LINC00473 served as a sponge of miR-195-5p, while our study demonstrated that LINC00473 not only sponged miR-195-5p but also negatively regulated miR-195-5p expression. Nevertheless, the detailed mechanism responsible for LINC00473-regulated miR-195-5p expression in prostate cancer remains to be explored in the future.

MiRNAs are a kind of small non-coding RNAs with approximately 19–24 nucleotides. These small RNAs take part in post-transcription of target genes by binding to the 3′-untranslated region (3′-UTR) of downstream target genes. Increasing studies suggested that miRNAs modulated gene expression in cell growth, proliferation and apoptosis. For example, miR-875-5p inhibits the cell invasion by targeting SATB2 in lung cancer [27]. MiRNA-876-5p regulates tumor development by binding with BCL6 corepressor like 1 in hepatocellular carcinoma [28]. MiR-195-5p has been identified to interact with NOB1, NOTCH2 and CCNE1 in several cancers [29–31], while miR-195-5p has not been investigated in prostate cancer. In our investigation, miR-195-5p specifically bound with 3′-UTR of SEPT2. What’s more, the SEPT2 expression was negatively regulated by miR-195-5p but positively regulated by LINC00473. All in all, LINC00473 served as the ceRNA against miR-195-5p to up-regulate SEPT2.

JAK-STAT3 pathway exists widely in kinds of cells, serving as a signal transduction pathway [32]. Moreover, JAK-STAT3 pathway was reported to be associated with cell proliferation and migration in cancers [33]. The main proteins in JAK-STST3 pathway were JAK and STAT3, and the activation of JAK-STST3 pathway was caused by phosphorylation of JAK and STST3 [34]. A previous study demonstrated that cAMP-PKA pathway regulates the expression of LINC00473 through STAT3 phosphorylation in human endometrial stromal cells [35]. Interestingly, current exploration discovered that the activation effect of LINC00473 on this pathway through targeting SEPT2 expression. However, how LINC00473/SEPT2 pathway could realize their regulation on JAK/STAT3 signaling pathway needs to be further elucidated.

This research is the first to explore the role and regulatory mechanism of LINC00473 in prostate cancer, and other mechanisms of LINC00473 in thyroid cancer deserve a further exploration in the future. All in all, our exploration verified that LINC00473 contributes to cell proliferation via JAK-STAT3 signaling pathway by regulating miR-195-5p/SEPT2 axis in prostate cancer, presenting a possible solution for treating patients with prostate cancer.

## Supplementary Material

Supplementary Figure S1 and S2

## Abbreviations

ceRNA: competing endogenous RNA
EdU: 5-ethynyl-2′-deoxyuridine
lncRNA: long noncoding RNA
miRNA: microRNA
RIP: RNA immunoprecipitation
