# Supplementary material for: The long non-coding RNA LINC00473 contributes to cell proliferation via JAK-STAT3 signaling pathway by regulating miR-195-5p/SEPT2 axis in prostate cancer
Source: Biosci Rep. 2020 Sep 16;40(9):BSR20191850. doi: 10.1042/BSR20191850 (PMC7494984; doi:10.1042/BSR20191850)
Supplement: Supplementary Figure S1 and S2 [file BSR-2019-1850_supp.pdf]

Supplementary file 1

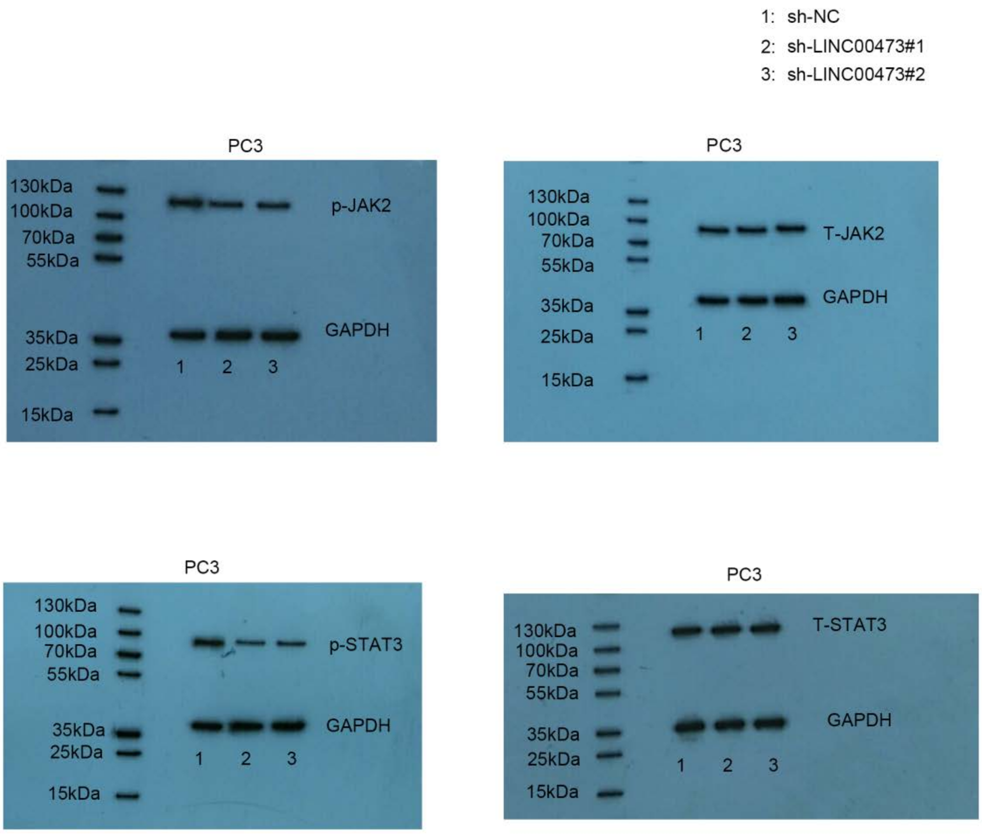

Supplementary file 1 The original protein bands for the data shown in the left panel of Figure 1E.

Supplementary file 2

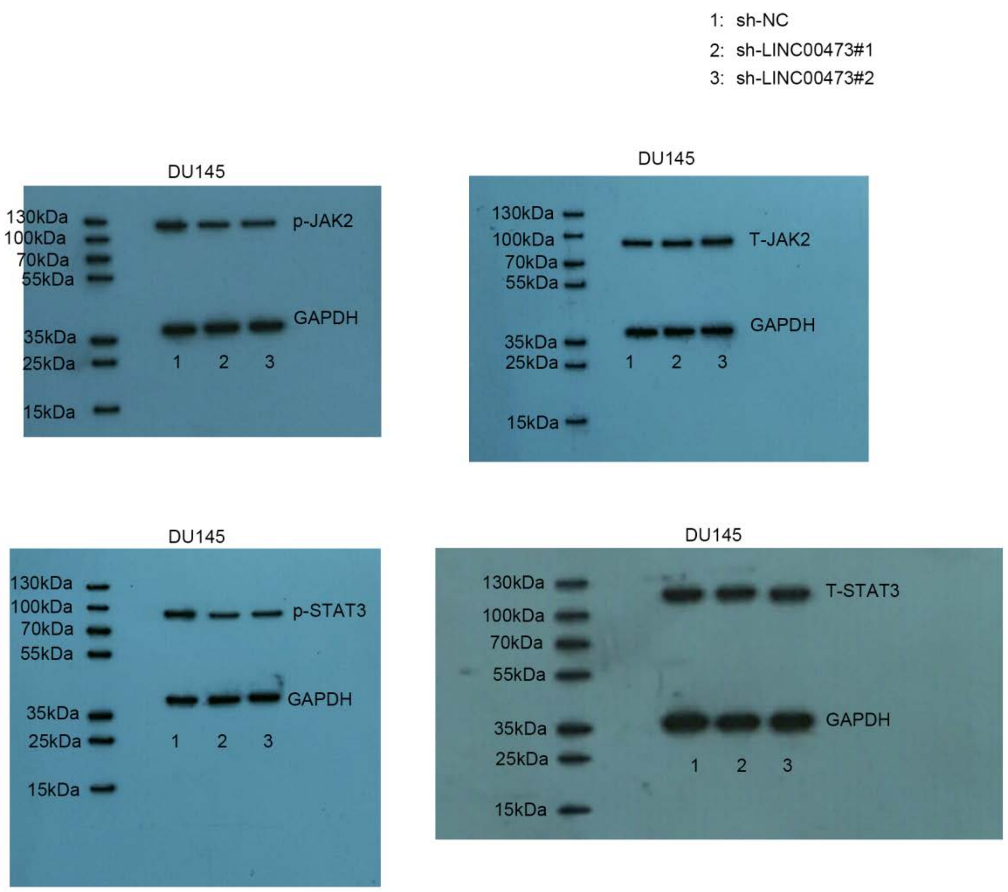

Supplementary file 2 The original protein bands for the data shown in the right panel of Figure 1E.
